# The Alcoholic Poison Beverage Habit—Two Great Rulers Awakened to Its Peril

**Published:** 1914-03

**Authors:** 


The Alcoholic Poison Beverage Habit—Two Great Rulers Awakened to Its Peril.—This year's announcement of the defensive attitude of President Wilson, with his associate Vice-President Marshall, also his great Premier W. J. Bryan, of His Majesty Emperor William of Germany, and later the Pope, against alcoholic beverage poisons is commendably significant of the awakening of our wise men in power to the peril to governments and people of the alcoholic popular drink habit. The danger of this blood, brain and general nerve cell poison is no longer seen afar off, nor as a mirage of the mind by "temperance kranks," but as near and real. Since the convincing revelations of the biochemical laboratory, the perils of alcoholic beverage poison are no longer seen from a distance or as through a glass darkly, but with illumined vision as to the destruction, plain and fatal, in the inebriate cup. The alcoholic canteen—the saloon for intoxicants will, if Americans are wise, be kept away from army and navy headquarters, even though camp and navy harbor neighborhoods must be more strictly patrolled. Soldiers, seamen and government officials should be always sober, and so should the American people. A sober nation, army and navy included, is safety for the nation.—*Alienist and Nenrologist.*

